# *Astragalus* saponins and its main constituents ameliorate ductular reaction and liver fibrosis in a mouse model of DDC-induced cholestatic liver disease

**DOI:** 10.3389/fphar.2022.965914

**Published:** 2022-10-20

**Authors:** Linzhang Zhang, Yonghong Hu, Shenglan Qi, Congcong Zhang, Qun Zhou, Dingqi Zhang, Yongping Mu, Hua Zhang, Gaofeng Chen, Ping Liu, Jiamei Chen, Wei Liu

**Affiliations:** ^1^ Key Laboratory of Liver and Kidney Diseases (Ministry of Education), Shanghai Key Laboratory of Traditional Chinese Clinical Medicine, Institute of Liver Diseases, Shuguang Hospital Affiliated to Shanghai University of Traditional Chinese Medicine, Shanghai, China; ^2^ The MOE Key Laboratory for Standardization of Chinese Medicines and the SATCM Key Laboratory for New Resources and Quality Evaluation of Chinese Medicines, Institute of Chinese Materia Medica, Shanghai University of Traditional Chinese Medicine, Shanghai, China

**Keywords:** astragalus saponins, DDC, cholestatic liver disease, astragaloside I, cycloastragenol

## Abstract

Cholestatic liver disease (CLD) is a chronic liver disease characterized by ductular reaction, inflammation and fibrosis. As there are no effective chemical or biological drugs now, majority of CLD patients eventually require liver transplantation. Astragali radix (AR) is commonly used in the clinical treatment of cholestatic liver disease and its related liver fibrosis in traditional Chinese medicine, however its specific active constituents are not clear. Total astragalus saponins (ASTs) were considered to be the main active components of AR. The aim of this study is to investigate the improvement effects of the total astragalus saponins (ASTs) and its main constituents in cholestatic liver disease. The ASTs from AR was prepared by macroporous resin, the content of saponins was measured at 60.19 ± 1.68%. The ameliorative effects of ASTs (14, 28, 56 mg/kg) were evaluated by 3, 5-Diethoxycarbonyl-1, 4-dihydrocollidine (DDC)-induced CLD mouse model. The contents of hydroxyproline (Hyp), the mRNA and protein expression of cytokeratin 19 (CK19) and α-smooth muscle actin (α-SMA) in liver tissue were dose-dependently improved after treatment for ASTs. 45 astragalus saponins were identified in ASTs by UHPLC-Q-Exactive Orbitrap HRMS, including astragaloside I, astragaloside II, astragaloside III, astragaloside IV, isoastragaloside I, isoastragaloside II, cycloastragenol, etc. And, it was found that ductular reaction in sodium butyrate-induced WB-F344 cell model were obviously inhibited by these main constituents*.* Finally, the improvement effects of astragaloside I, astragaloside II, astragaloside IV and cycloastragenol (50 mg/kg) were evaluated in DDC-induced CLD mice model. The results showed that astragaloside I and cycloastragenol significantly improved mRNA and protein expression of CK19 and α-SMA in liver tissue. It suggested that astragaloside I and cycloastragenol could alleviate ductular reaction and liver fibrosis. In summary, this study revealed that ASTs could significantly inhibit ductular reaction and liver fibrosis, and astragaloside I and cycloastragenol were the key substances of ASTs for treating cholestatic liver disease.

## Introduction

Cholestasis is a pathological condition in which bile formation, secretion and excretion are impaired inside and outside the liver due to various reasons, and bile flow cannot flow properly into the duodenum and enters the bloodstream (Hasegawa et al., 2021). Hepatobiliary diseases in which cholestasis is the main manifestation of liver lesions for various reasons are named cholestatic liver disease, which itself can further aggravate liver damage (Brevini et al., 2020; Stättermayer et al., 2020). Ductular reaction and liver fibrosis are typical pathological manifestations of cholestatic liver disease (Kummen et al., 2021). There are no definitive data on the incidence of cholestatic liver disease. A study about patients with initially diagnosed chronic liver disease showed that cholestasis occurred in 882 of 2,520 patients (35%) with chronic liver disease and was more likely to be seen in PBC and PSC (Wagner and Fickert, 2020). UDCA is the common clinical drug for PBC, but there is still a significant unmet need for treatment of patients with poor response, and there are no chemical or biological drugs for PSC, liver transplantation is currently the only effective treatment (Weismüller et al., 2017), it is an urgent need for developing new drugs.

Astragali radix (AR) has been used commonly in traditional Chinese medicine for more than 2000 years (Yu et al., 2018). It is the dried root of *Astragalus membranaceus* (Fisch.) Bge. var. mongholicus (Bge.) Hsiao. or *Astragalus membranaceus* (Fisch.) Bge. Pharmacological studies and clinical practices indicate that AR had various medicinal effects including anti-inflammatory, anti-cancer, hepatoprotective, cardioprotective, neurological, and antiviral. It is widely used for the treatment of anemia, liver fibrosis and cirrhosis, allergy, chronic fatigue, loss of appetite, uterine prolapse and other diseases (Fu et al., 2014; Zhang L. et al., 2017). *Astragalus* saponins (ASTs) are considered to be the main active components of AR, more than 200 AR compounds have been independently reported (Guo et al., 2019). Studies have shown that AR and its components had good protective effects against various liver disease models induced by carbon tetrachloride (CCl_4_), dimethylnitrosamine (DMN), and α-naphthyl isothiocyanate (ANIT) (Vitcheva et al., 2013; Zhou et al., 2016; Qiu et al., 2021). And ASTs are effective in improving cardiovascular diseases, diabetes, neurological degeneration, and other diseases (Xia et al., 2020; Meng et al., 2021; Su et al., 2021). However, few results have reported the effects of ASTs on cholestatic liver disease. Therefore, we investigate the effects and active constituents of ASTs in cholestatic liver disease.

In this study, ASTs are assessed *in vivo* by 3,5-diethoxycarbonyl-1,4-dihydrocollidine (DDC)-induced CLD mouse model, which is a classical animal model of cholestatic liver disease induced by toxicant (Fickert et al., 2007). Meanwhile, the chemical profiling of ASTs and absorbed chemical constituents are analysed by ultra-high-performance liquid chromatography-Q exactive hybrid quadrupole orbitrap high-resolution accurate mass spectrometriy (UHPLC-Q-Exactive Orbitrap HRMS) after oral aderministration of ASTs. Further, the effect of inhibiting ductular reaction of main constituents is evaluated by sodium butyrate (SB)-induced WB-F344 cell model *in vitro*. Finally, the improvement effects of main absorbed chemical constituents (astragaloside I, astragaloside II, astragaloside IV and cycloastragenol) are evaluated by DDC-induced CLD mice model. The results clarify the major active constituents of ASTs for the treatment of cholestatic liver disease, and with a view to provide references for clinical application and further research of AR.

## Materials and methods

### Materials and reagents

DDC and SB are purchased from Sigma-Aldrich (STBK3562, B5887, St. Louis, MO, United States). The DDC diet ingredients were normal chow (which contains 203.5 g/kg crude protein, 43.1 g/kg crude fat, 48.9 g/kg crude fiber, 91.8 g/kg moisture, 59.3 g/kg ash, 14.8 g/kg calcium, 9.1 g/kg phosphorus. Energy was about 3,600 kcal/kg) and 0.1% DDC. The DDC diet was prepared by Fanbo biotechnology Co. (M2011, Jiangsu, China). Astragaloside I, astragaloside II, astragaloside III, astragaloside IV, isoastragaloside I, isoastragaloside II and cycloastragenol are purchased from Chengdu Biopurify. Co. Obeticholic acid and D101 macroporous resin are purchased from Dalian Melan Co. DMEM and FBS are purchased from Gibco Life Technology (Gibco Invitgen Corporation, Barcelona, Spain). Alanine aminotransferase (ALT), aspertate aminotransferase (AST), total bilirubin (TBiL), direct bilirubin (DBiL), indirect bilirubin (IBiL) kits, hematoxylin eosin (H&E) staining kits and hydroxyproline (Hyp) kits are purchased from Nanjing Jiancheng Institute of Biological Engineering. The reverse transcriptase assay kit, Trizol reagent, and SYBR Green Realtime PCR MASTER Mix are purchased from Takara Biotech Co. RIPA lysis buffer and BCA protein concentration assay kits are purchased from Beyotime BioLtd. The antibodies cytokeratin 19 (#10712-1-AP), α-smooth muscle actin (#ab124964) and GAPDH (#60004-1-Ig) are purchased from Proteintech (Wuhan, China). Astragali radix (AR) (No. 220120) was purchased from Shanghai Hongqiao Traditional Chinese Medicine Decoction Piecess Co., Ltd. (Shanghai, China), and the herb was authenticated as *Astragalus membranaceus* (Fisch.) Bge. var. mongholicus (Bge.) Hsiao. by associate professor LIU Wei, from the Institute of Liver Diseases, Shuguang Hospital Affiliated to Shanghai University of Traditional Chinese Medicine.

### Animals

Three batches of male C57/BL6J mice, 48, 6, and 56 mice, with a body mass of 20 ± 2 g, are purchased from Beijing Vital River Laboratory Animal Co. The animals are housed in the Experimental Animal Center of Shanghai University of Traditional Chinese Medicine. The experiments are approved by the Experimental Animal Ethics Committee of Shanghai University of Traditional Chinese Medicine with the following ethics number: PZSHUTCM201016007. The ambient temperature is 22 ± 2°C, and the duration of daylight and darkness are 12 h each, with free access to water and food.

### Preparation of astragalus saponins

The preparation procedures of ASTs from AR by macroporous resin column chromatography are described in previous report (Chu et al., 2022). Dried AR (2 kg) is sheared into segments and extracted with 20 L of 70% ethanol (*v*/*v*) thrice in reflux, each for 2 h. Extracts are combined, filtered, and concentrated under reduced pressure at 45°C to afford 4 L concentrated extract of AR. The concentrated extract is separated and prepared ASTs by macroporous resin column chromatography (4 kg, column volume is about 3 L), being eluted with a gradient system of water - ethanol (100:0, 70:30, 10:90), 20 L per elution segment. Finally, the 90% ethanol fraction (mainly contains saponins) is collected, and evaporated under reduced pressure with the rotary evaporator at 45°C and the concentrate freeze-dried to yield solid material (10.23 g, extract yield is 0.51% from AR). The average content of total saponins in the preparation ASTs is determined as 60.19 ± 1.68% using the method reported in the literature (Kong et al., 2010). Identification and characterization of chemical constituents from ASTs is using UHPLC-Q-Exactive Orbitrap HRMS (Supplementary Table S1).

### Intervention effects of ASTs on 0.1% DDC-induced mouse model

Forty-eight mice are randomly divided into normal control group (*n* = 8) and model group (*n* = 40). The normal control group is fed normal chow, the model group is fed chow containing 0.1% DDC. After 4 weeks of feeding, the 40 model mice are further randomly divided into five groups of eight mice each: CLD model group, 14 mg/kg ASTs group, 28 mg/kg ASTs group, 56 mg/kg ASTs group (the dose is used human equivalent dose [65 kg, 15, 30, 60 g recipe per day]), and 10 mg/kg obeticholic acid group (positive control). The normal and model groups are gavaged 0.4% CMC-Na solution from week five to week eight once a day, and the treatment groups are given the corresponding drug by gavage once a day. At the end of week 8, the mice are executed, blood and liver specimens are collected for further experiments.

### Characterization of the absorbed chemical constituents in mice serum and liver after oral administration of ASTs

After 7 days of acclimatization, six male mice are randomly divided into two groups (3 mice for each group). ASTs group is oral administered 56 mg/kg ASTs solution; control group is oral administered 0.4% CMC-Na solution. All animals are fasted overnight before the experiment and have free access to water. One hour after once oral administration of ASTs, all mice are anesthetized. Blood samples are collected from the abdominal aorta, and liver tissues are quickly removed. Serum samples are obtained by centrifuged at 8,000 × g for 10 min at 4°C. All samples are stored at −80°C until analysis. The serum samples (200 μL) are added five times methanol (1 ml), vortexed and then, centrifuged at 12,000 × g for 10 min. The supernatant (960 μL) is dried with nitrogen gas. The residue is redissolved in 80 μL 10% methanol, vortexed and then, centrifuged at 12,000 × g for 10 min, and the supernatant is used in the UHPLC-Q-Exactive Orbitrap HRMS analysis. The 100 mg liver tissue is homogenized in 1 ml methanol on ice, the homogenate of them is centrifuged at 4°C at 12,000 × g for 10 min, The supernatant (800 μL) is dried with nitrogen gas. The residue is redissolved in 80 μL 10% methanol, vortexed and then, centrifuged at 12,000 × g for 10 min, and the supernatant is used in the UHPLC-Q-Exactive Orbitrap HRMS analysis.

### The inhibitory effects of the main chemical constituents of ASTs on ductular reaction in sodium butyrate-induced WB-F344 cell model

The cell viability assay of main chemical constituents of ASTs is evaluated by CCK-8. WB-F344 cells are plated at 5 × 10^3^ cells/well in 96-well plates and cultured in culture medium containing 10% FBS. After 24 h, the cells are incubated with ASTs and its seven components at different concentrations (ASTs at concentrations of 10, 20, 40, 60, 80 and 100 μg/ml. Astragaloside Ⅰ, astragaloside Ⅱ, astragaloside Ⅲ, astragaloside Ⅳ, isoastragaloside Ⅰ, isoatragaloside Ⅱ and cycloastragenol at concentrations of 6.25, 12.5, 25, 50 and 100 μM, respectively for 24 h). Subsequently, all wells are added into CCK-8 solution. After 1 h incubation, the absorbance is read at 450 nm, and the cell viability is calculated.

The effect of main constituents of ASTs is evaluated in sodium butyrate-induced WB-F344 cell model. WB-F344 cells are inoculated in 6-well plates with 3×10^4^ cells and cultured in DMEM medium containing 10% FBS. The cells are divided into DMSO control group, SB (3.75 µM) group, SB + astragaloside I (10 µM) group, SB + astragaloside II (10 µM) group, SB + astragaloside Ⅲ (10 µM) group, SB + astragaloside IV (10 µM) group, SB + isoastragaloside Ⅰ (10 µM) group, SB + isoatragaloside Ⅱ (10 µM) group, SB + cycloastragenol (10 µM) group and SB + ASTs (10 μg/ml) group. Drug-containing medium and fresh medium are replaced after 2 days. All cells are maintained for 4 days and collected for mRNA analysis by qRT-PCR.

### Intervention effects of astragaloside I, astragaloside II, astragaloside IV, cyclogalactol on 0.1 %DDC-induced CLD mouse model

Fifty-six mice are randomly divided into normal control group (*n* = 8) and CLD model group (*n* = 48). The normal control group is fed normal chow, the model group is fed chow containing 0.1% DDC. After 4 weeks of feeding, the 48 model mice are further randomly divided into six groups of eight mice each: CLD model group, 50 mg/kg astragaloside I group, 50 mg/kg astragaloside II group, 50 mg/kg astragaloside IV group, 50 mg/kg cycloastragenol group, and 56 mg/kg ASTs group. From week five to week 8, normal group and model group are given 0.4% CMC-Na solution by gavage once a day, and drug groups are given the corresponding drug by gavage once a day. The mice are executed at the end of week 8, blood and liver specimens are collected for subsequent experiments.

### Serum biochemistry and pathology analysis

Serum samples are thawed at 4°C one night in advance and placed in the autoanalyzer according to the labeling order, followed by adding the corresponding assay reagents to the assay vials to detect serum ALT, AST, TBiL, DBiL and IBiL levels. Liver tissues are fixed in neutral formalin, embedded in paraffin, sectioned, stained with H&E and Sirius Red. They are processed for quantitative analysis of the positive area of SR staining by using Image Analysis.

### Hepatic hydroxyproline content assays

The liver tissue (50.0 mg) is weighed into a 1.5 ml centrifuge tube. Each sample is added to 1 ml of hydrolysate, then incubated in a water bath at 95°C for 10 min*.* After cooling to room temperature, the pH is adjusted to 6.0–6.8. The supernatant (4 ml) is added to 30 mg of activated carbon and centrifuged. Then each tube is added to agents I, II and III respectively. 200 μL of the mixture is used to determine the OD value at 550 nm, and the hyp content of each sample is calculated.

### Immunohistochemistry assays

Paraffin tissue sections are dewaxed, dehydrated and antigen repair is performed with 4% citrate buffer. Sections are incubated with 3% H_2_O_2_ for 20 min and 10% goat serum for 30 min. Anti-CK19 (1:500) and anti-α-SMA (1:1,000) are incubated at 4°C overnight. Sections are incubated with HRP-labeled secondary antibody in the next day. The tissue sections are stained by 3, 3-diaminobenzidine (DAB) and H&E staining. The positive area of tissue staining is quantified using Image Analysis.

### Quantitative real-time PCR analysis

Mouse liver tissue and WB-F344 cells are lysed with Trizol reagent. Then chloroform, isopropanol, 75% ethanol and anhydrous ethanol are added respectively. Finally DEPC water is added. The NanoVue concentration detector is applied to detect the RNA concentration. Total RNA is reverse transcribed using reverse transcription kit to obtain cDNA. 384-well PCR plates are spiked, and the corresponding indexes are detected by Real-time PCR method. The expression of each mRNA is calculated by 2-^ΔΔ^Ct method. The amount of mRNA is normalized using GAPDH as an internal standard. qRT-PCR primer sequences are shown in Table 1.

**TABLE 1:** qRT-PCR primer sequences.

Species	Gene	Forward primer (5′–3′)	Reverse primer (5′–3′)
Mouse	CK19	GGA​AGG​TGA​TAT​TGT​GTT​CGC​C	CTA​TGG​TCT​CCT​CTG​TAG​AAG​GC
Mouse	CK7	AGG​AAC​AGA​AGT​CAG​CCA​AGA​G	GCA​ACA​CAA​ACT​CAT​TCT​CAG​C
Mouse	α-SMA	CAC​TGT​AAC​TGG​GGG​CAA​CT	CAC​TTC​TTG​TCA​GCG​TCG​AA
Mouse	Col1A1	GAT​TCC​CTG​GAC​CTA​AAG​GTG​C	AGC​CTC​TCC​ATC​TTT​GCC​AGC​A
Mouse	GAPDH	AGC​CAG​AGC​TGT​GCA​GAT​GA	GCA​GGC​TGG​CAT​TTG​TGG​TT
Rat	CK19	CCC​CAA​AGG​GAT​GAG​AAG​TT	CAC​TTG​GTG​GTT​TGC​TAC​GA
Rat	GAPDH	CCA​TCA​ACG​ACC​CCT​TCA​TT	GAC​CAG​CTT​CCC​ATT​CTC​AG

### Western blot analysis

The liver tissue (40.0–60.0 mg) is weighed and added to 1 ml of protein lysate. The lysed tissues are centrifuged at 12,000 rpm for 30 min, and the protein concentration is measured by BCA protein assay kit. Equal amounts of protein samples are separated by SDS-PAGE and transferred to polyvinylidene fluoride (PVDF) membranes. The membranes are blocked for 1 h at room temperature in Western blocking solution. The primary antibody is incubated at 4°C overnight and the secondary antibody is incubated at room temperature for 1 h in the following day. The primary antibody dilutions are incubated with anti-CK19 (1:1,000), anti-α-SMA (1:1,000), GAPDH (1:5,000) and secondary antibody (1:1,000). The target bands are scanned and compared with GAPDH bands for statistical analysis.

### Statistical analysis

SPSS 23.0 software is used to statistically analyze the data. The data is expressed as mean ± standard deviation (SD), and *p* < 0.05 is considered statistically significant. One-way ANOVA analysis is used for the comparative analysis of multiple groups, and Student’s t-test is used for the statistical comparison between two groups.

## Results

### ASTs improves serum biochemistry and collagen deposition in DDC-induced CLD mice

**FIGURE 1:**
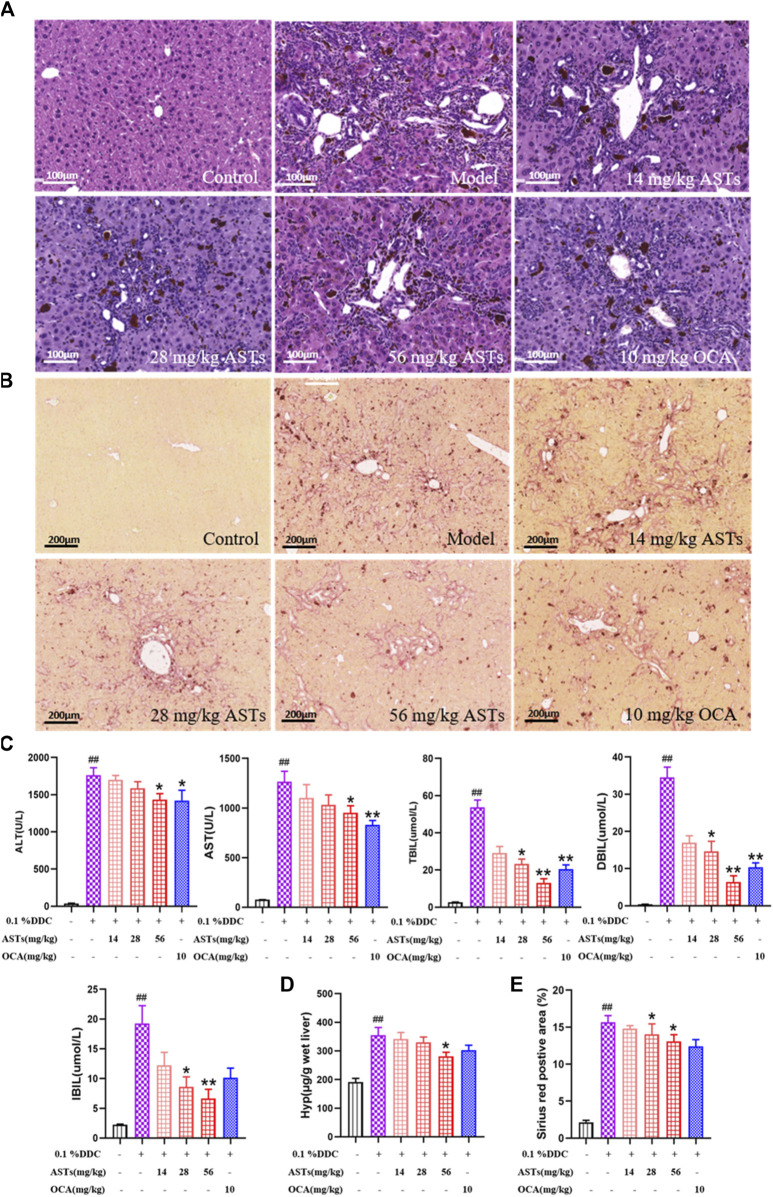
Effects of ASTs on serum biochemistry and collagen deposition in 0.1% DDC-induced CLD mice. **(A,B)** Representative images of H&E staining (×20x) (scale bar 100 μm), sirius red staining (×10x) (scale bar 200 μm) are performed to observe the histopathological characteristics of liver tissues after the treatment of ASTs and OCA. **(C)** Measurement of serum ALT, AST, TBiL, DBiL and IBiL levels in mice are deceted. **(D)** Hyp levels in liver tissues are determined, **(E)** Area (%) of positive sirius red is assessed by quantitative imaging of sirius red staining. ^##^ *p* < 0.01 vs. the normal group. ^∗∗^ *p* < 0.01 and ^∗^ *p* < 0.05 vs. 0.1% DDC group. Values were expressed as mean ± SD.

Normal morphology and regular arrangement of hepatocytes in the normal group are shown in H&E staining (Figure 1A). Compared with the normal group, porphyrin deposition, cell necrosis and inflammatory cell infiltration are increased in the model group, and the number of hyperplastic bile ducts is increased. Compared with the model group, the ductular reaction and inflammation are reduced to different degrees in ASTs-treated groups. In addition, sirius red staining shows that (Figure 1B), collagen deposition is rarely seen in the normal group, and compared with the normal group, a large number of collagen are deposited around the hyperplastic bile ducts in the model group, while collagen deposition is alleviated in ASTs-treated groups.

Serum ALT, AST, TBiL, DBiL and IBiL levels are significantly increased in the model group (*p* < 0.01) than these in the normol group, the elevation of each index is remarkablely reduced after ASTs and obeticholic acid treatment (*p* < 0.01 or *p* < 0.05) (Figure 1C). In addition, liver Hyp content and Sirius red positive area are significantly reduced after ASTs treatment (*p* < 0.05). However, there is no significant difference between the obeticholic acid group and the model group (Figures 1D,E).

### ASTs regulates the expressions of CK19, CK7, α-SMA, COL1A1 in DDC-induced CLD mice

**FIGURE 2:**
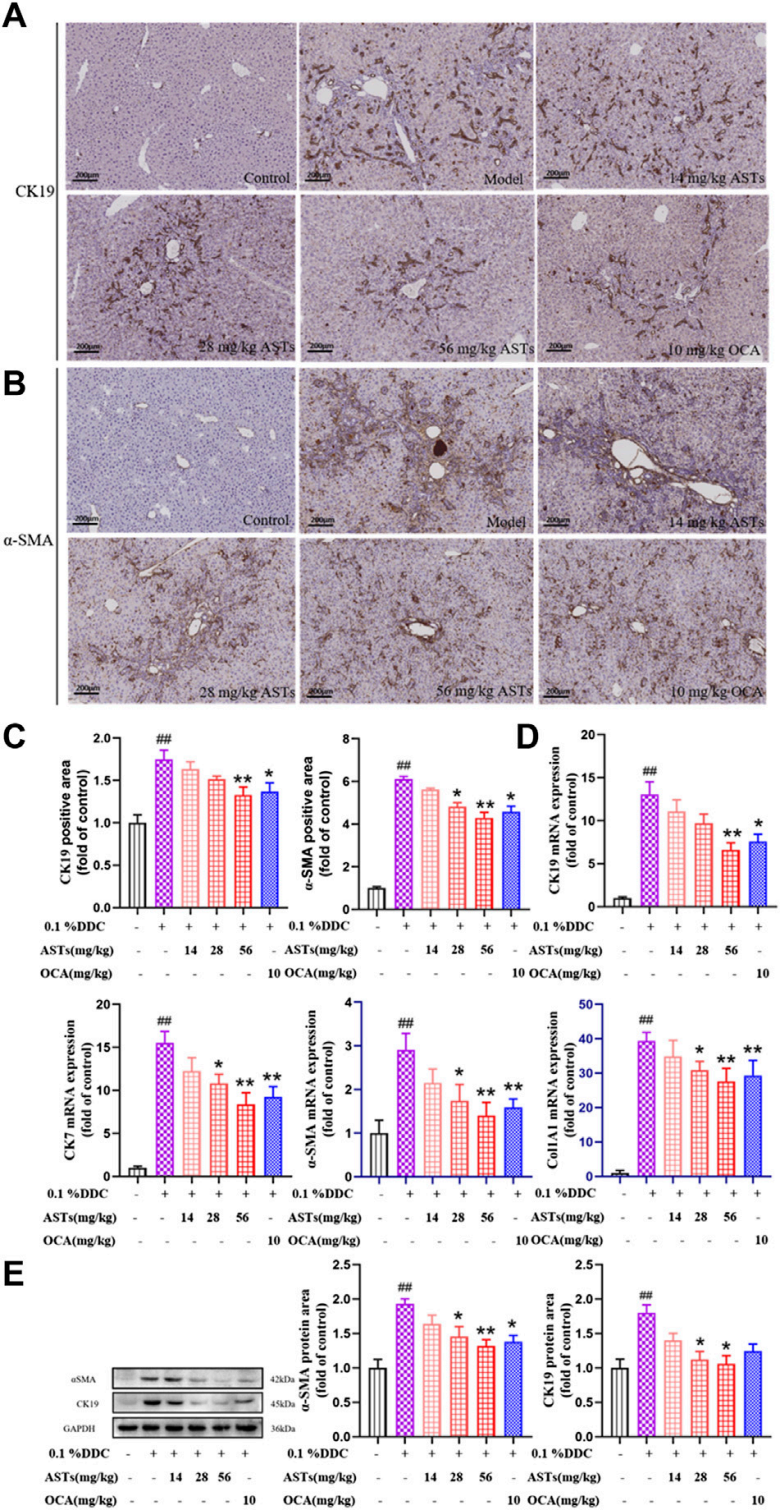
Effects of ASTs on the expressions of CK19, CK7, α-SMA, Col1A1 in 0.1% DDC-induced CLD mice. **(A,B)** Immunohistochemical method is used to detect the effects of ASTs and OCA on hepatic α-SMA and CK19 expressions in the liver (×10x) (scale bar 200 μm). **(C)** α-SMA and CK19 positive areas are assessed by immunohistochemical quantification. **(D)** Effects of ASTs on the gene expressions of CK19, CK7, α-SMA and Col1A1 are evaluated by qRT-PCR, and normalized against GAPDH level. **(E)** Protein expressions of α-SMA and CK19 in liver are assessed by western blot and normalized to GAPDH protein. ^##^ *p* < 0.01 vs*.* the normal group. ^∗∗^ *p* < 0.01 and ^∗^ *p* < 0.05 vs*.* 0.1% DDC group. Values were expressed as mean ± SD.

Hepatic α-SMA and CK19 are abundantly expressed in the liver of the model group by immunohistochemical staining (Figures 2A,B). Compared with the normal group, the positive areas of α-SMA and CK19 are significantly higher in the model group (*p* < 0.01), and significantly attenuated in the ASTs-treated and obeticholic acid-treated groups (*p* < 0.01 or *p* < 0.05) (Figure 2C).

The mRNA expressions of CK19, CK7, α-SMA and Col1A1 are significantly increased in the model group (*p* < 0.01) (Figure 2D), while the expressions of CK19, CK7, α-SMA and Col1A1 are significantly reduced in the ASTs-treated group (*p* < 0.01 or *p* < 0.05). Likewise, the protein expressions of CK19 and α-SMA are consistent with the qRT-PCR results (Figure 2E).

### Identification of the chemical constituents in ASTs and the absorbed chemical constituents in mice serum and liver after oral administration of ASTs

**FIGURE 3:**
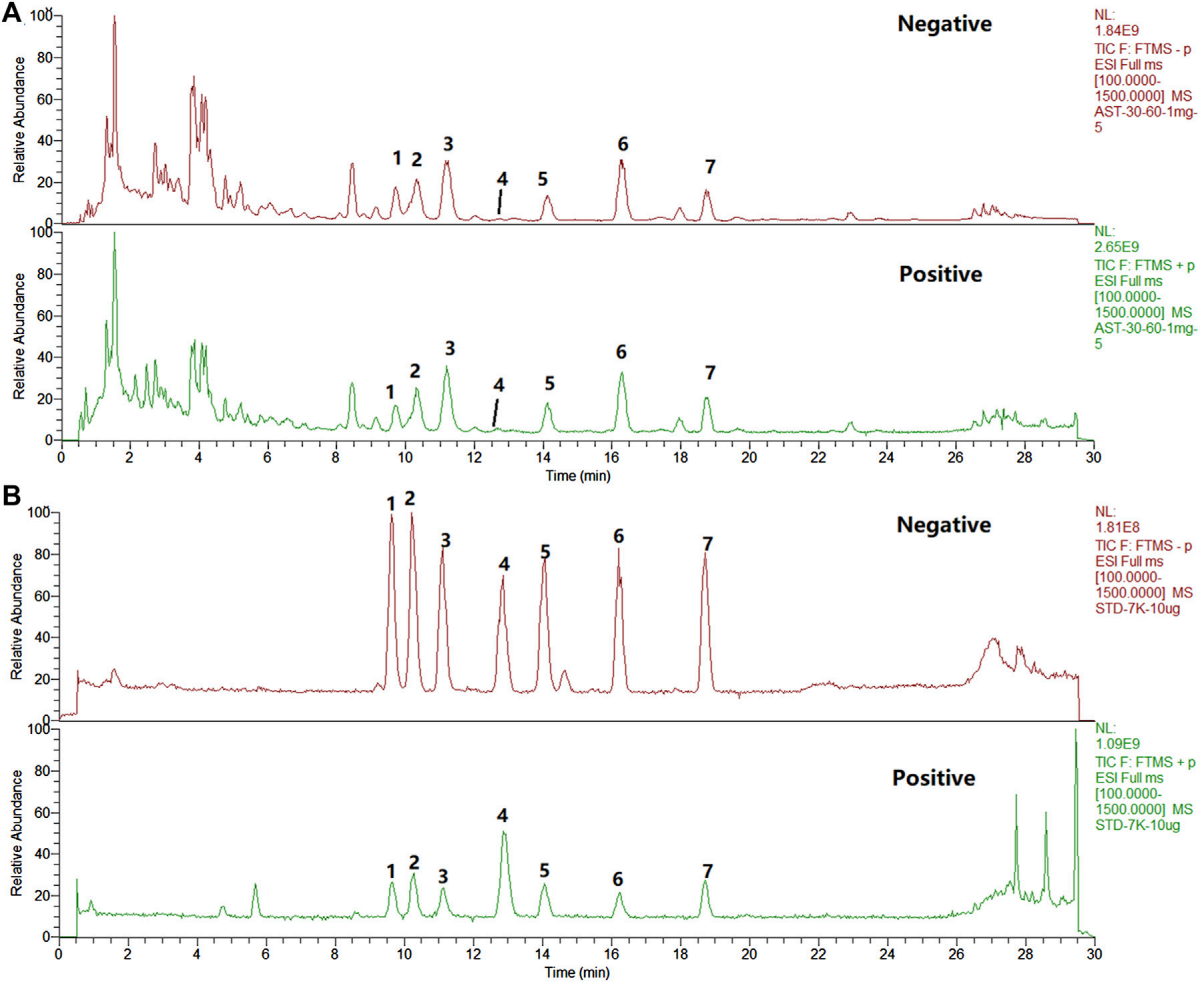
The total ion chromatograms (TICs) of the extract of ASTs and seven reference substances by UHPLC-Q-Exactive Orbitrap HRMS. **(A)**, ASTs solution; **(B)**, standard solution; 1, astragaloside III; 2, astragaloside IV; 3, astragaloside II; 4, cycloastragenol; 5, isoastragaloside II; 6, astragaloside I; 7, isoastragaloside I.

**FIGURE 4:**
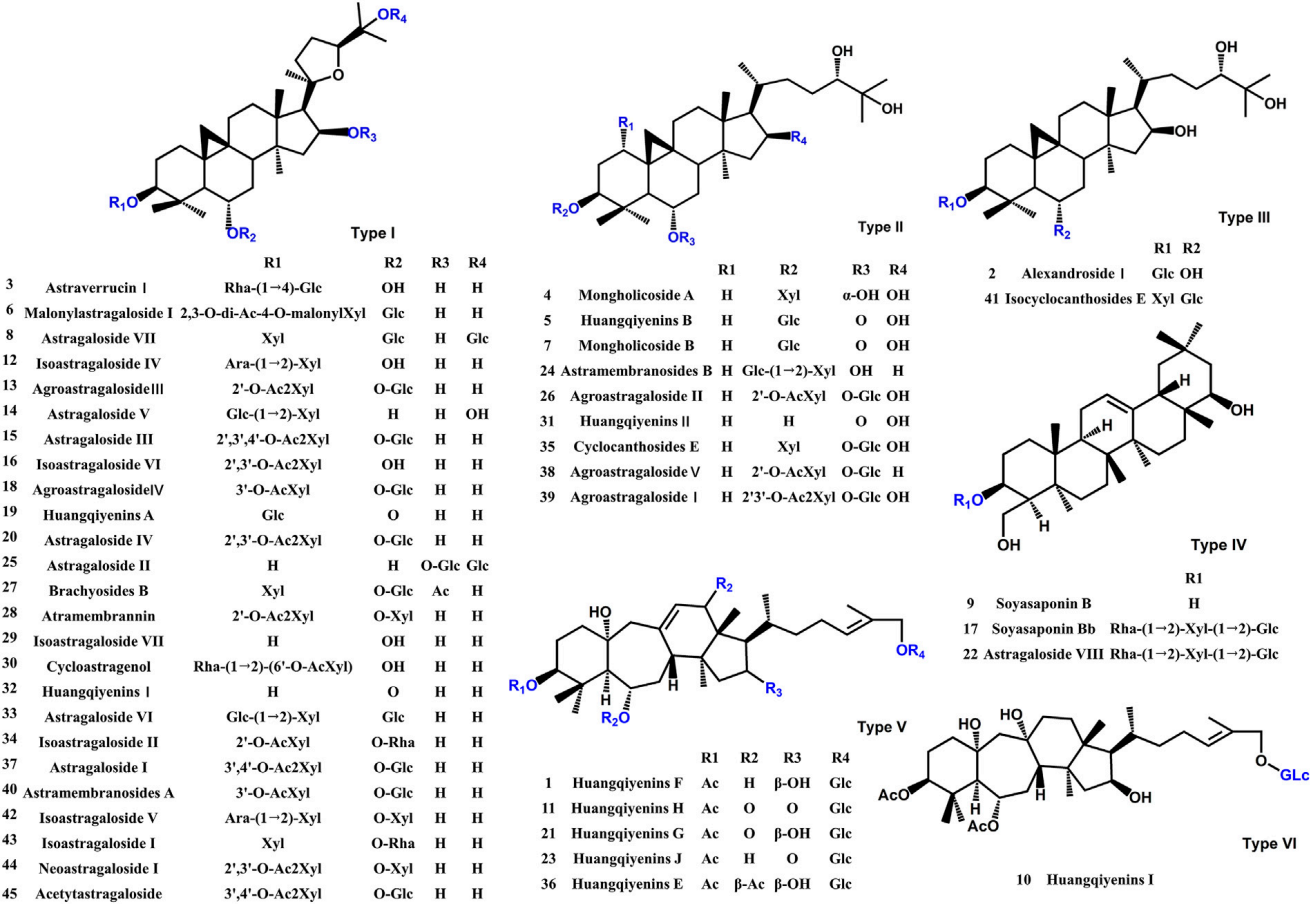
The chemical structures of identified *Astragalus* saponins.

A comprehensive analysis of the components in ASTs is performed by UHPLC-Q-Exactive Orbitrap HRMS under the above optimal conditions. The total ion chromatograms (TICs) of the ASTs and seven astragalus saponins standards in positive and negative ion mode are shown in Figure 3A. The calculated errors of the protonated molecular weights of all identified compounds are within 10 ppm. 45 kinds of astragalus saponins are identified or tentatively determined from the ASTs after careful comparison with the retention times and MS/MS profiles of standards, references and chemistry books. Detailed information on the identified chemical components in the ASTs, including retention times, precise molecular weights, MS/MS fragment ions and peak areas of each component are listed in Supplementary Table S1. And specific structures of identified 45 astragalus saponins are shown in Figure 4. The TICs of seven astragalus saponins reference standards in positive and negative ion mode are shown in Figure 3B, and the contents of astragaloside III, astragaloside IV, astragaloside II, cycloastragenol, isoastragaloside II, astragaloside I, and isoastragaloside I are 23.81 mg/g, 25.98 mg/g, 66.17 mg/g, 0.42 mg/g, 27.19 mg/g, 79.46 mg/g and 34.29 mg/g in the ASTs, respectively. The serum and liver samples collected from ASTs-treated mice at 1 h are collected for LC-MS analysis to detect the absorbed prototypes as more as possible. With the help of the key information of the constituents (as listed in Supplementary Table S1) and the identification of the chemical constituents in ASTs, astragaloside IV, astragaloside II, cycloastragenol, and astragaloside I are obviously detected in serum or liver after oral administration 56 mg/kg ASTs. Other saponins constituents are not detected in serum or liver, it may be related with the relatively low contents of them in ASTs.

### Effects of main chemical constituents of ASTs on SB-induced WB-F344 cell model

**FIGURE 5:**
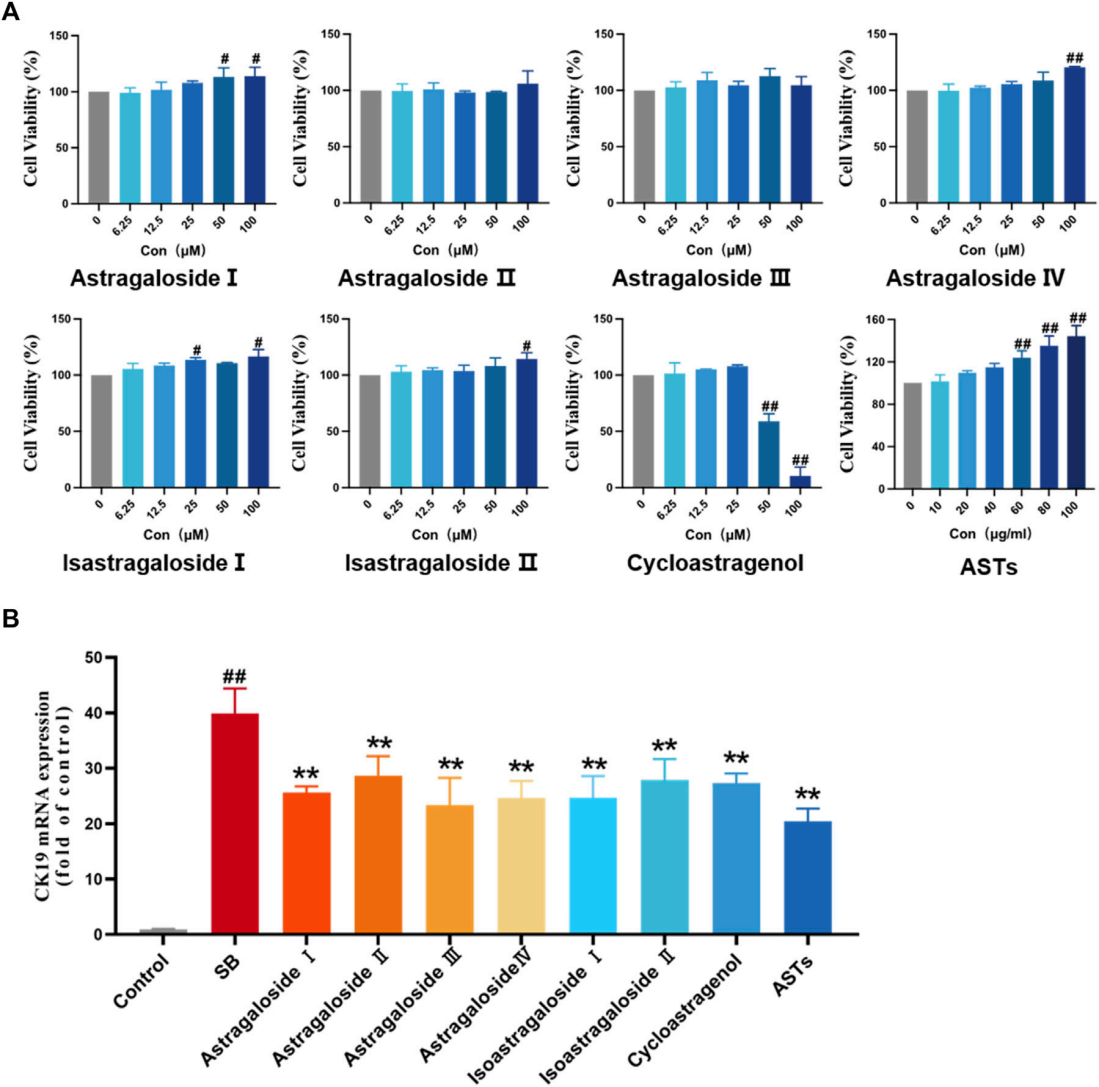
Effects of ASTs and main components in SB-induced WB-F344 cell model. **(A)** Cell viability is determined by CCK8 assay. **(B)** Expression of CK19 is determined by qRT-PCR. ^##^ *p* < 0.01 and ^#^ *p* < 0.05 vs*.* the control group. ^∗∗^ *p* < 0.01 vs*.* SB model group. Values were expressed as mean ± SD.

On the basis of effects of ASTs *in vivo* and the chemical analysis results of UHPLC-Q-Exactive Orbitrap HRMS, the effects of main seven chemical constituents of AST on SB-induced WB-F344 cell are evaluated. First, the cell viability is evaluated by CCK8 assay. Compared with the control group, cell viability is not significantly inhibited within 12.5 µM of these compound, but is obviously inhibited by isoastragaloside I treatment with 25 μM, astragaloside I and cycloastragenol treatment with 50 µM (*p* < 0.01 or *p* < 0.05) (Figure 5A). Consistent with the *in vivo* experiments, the expression of CK19 is evaluated *in vitro*. The elevated mRNA expression of CK19 in SB-induced WB-F344 cell is significantly decreased in these compounds (10 µM) and ASTs groups (10 μg/ml) (*p* < 0.01) (Figure 5B).

### Astragaloside I and cycloastragenol improve serum biochemistry and collagen deposition in DDC-induced CLD mice

**FIGURE 6:**
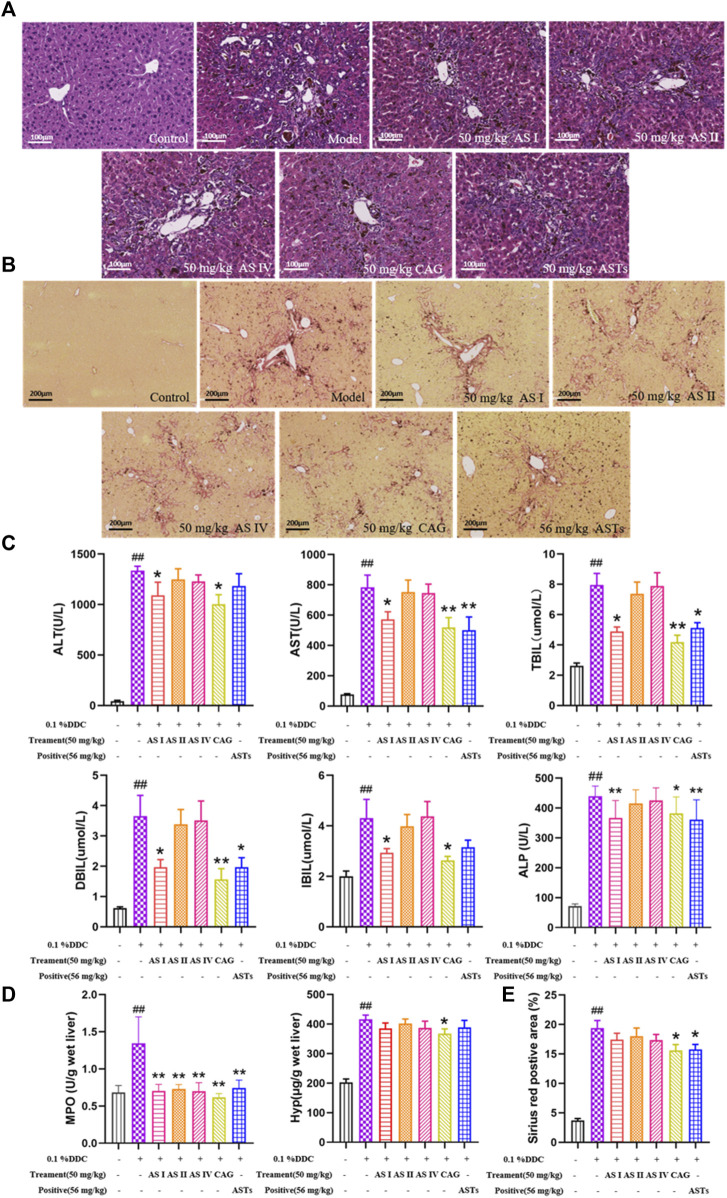
Effects of astragaloside I, astragaloside II, astragaloside IV and cyclogalactol on liver injury and collagen deposition of 0.1% DDC-induced mice. **(A,B)** Representative images of H&E staining of liver tissue (×20x) (scale bar 100 μm), sirius red staining (×10x) (scale bar 200 μm) are performed to observe the histopathological characteristics of liver tissues. **(C)** Measurement of serum ALT, AST, TBiL, DBiL, IBiL and ALP levels are deceted in mice. **(D)** MPO and Hyp content in liver tissues are determinated. **(E)** Area (%) of positive collagen is assessed by quantitative imaging of sirius red staining. ^##^ *p* < 0.01 vs*.* the normal group. ^∗∗^ *p* < 0.01 and ^∗^ *p* < 0.05 vs*.* 0.1% DDC group. Values were expressed as mean ± SD.

Normal morphology of hepatocytes without degeneration and necrosis are showed in the normal group by H&E staining (Figure 6A). Compared with the normal group, the ductular reaction phenomenons are increased in the model group, including bile duct dilatation, increase in the number of hyperplastic bile ducts, structural disorder, massive inflammatory cell infiltration, and focal necrosis of hepatocytes. The above phenomenons in four saponins and ASTs groups are improved in different degrees. Sirius red staining (Figure 6B) showed that a large amount of collagen fibers are deposited around the hyperplastic bile ducts. The collagen deposition is reduced in each treatment group, and the best improvement effect is observed in the astragaloside I, cycloastragenol and ASTs groups.

Serum ALT, AST, TBiL, DBiL, IBiL and ALP levels are significantly elevated in the CLD model mice compared with the normal mice (*p* < 0.01), and the elevation of each index is significantly reduced in the astragaloside I, cycloastragenol and ASTs group (*p* < 0.01 or *p* < 0.05) (Figure 6C). Liver MPO, liver Hyp content and sirius red positive area are significantly elevated in the model group (*p* < 0.01), and significantly alleviated in the cycloastragenol and ASTs group (*p* < 0.05) (Figures 6D,E).

### Astragaloside I and cycloastragenol supppressed the expressions of CK19, CK7, α-SMA, COL1A1, IL-1β, and IL-6 in DDC-induced CLD mice

**FIGURE 7:**
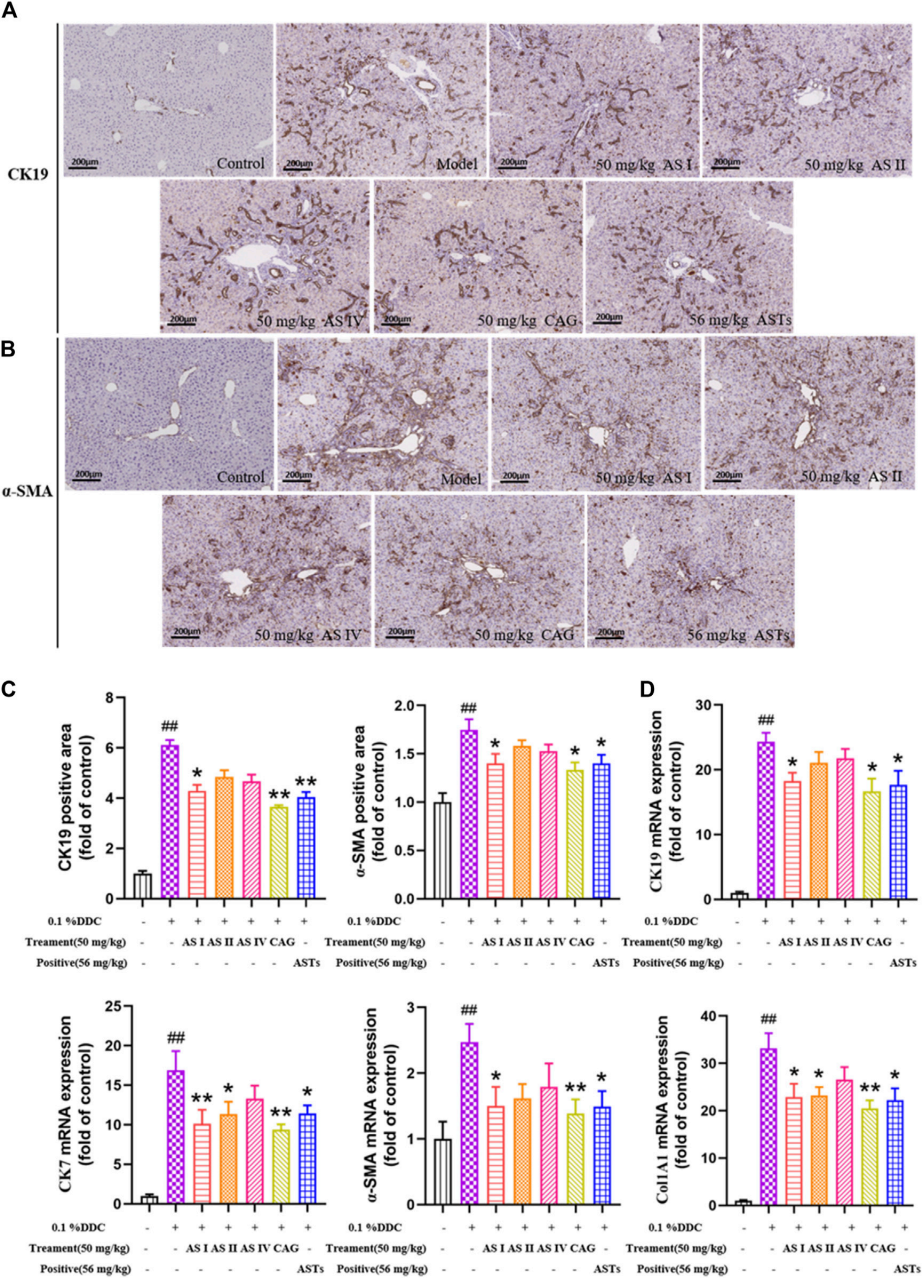
Effects of astragaloside I, astragaloside II, astragaloside IV and cyclogalactol on the expressions of CK19, CK7, α-SMA and Col1A1 in 0.1% DDC-induced CLD mice. **(A,B)** Immunohistochemical method is used to detect the effects of different astragalus saponins on hepatic α-SMA and CK19 protein expressions (×10x) (scale bar 200 μm). **(C)** Morphometric quantification of the α-SMA and CK19-positive area (%). **(D)** Effects of different astragalus saponins on the gene expressions of CK19, CK7, α-SMA and Col1A1 are evaluated by qRT-PCR, and normalized to GAPDH gene. ^##^ *p* < 0.01 vs*.* the normal group. ^∗∗^ *p* < 0.01 and ^∗^ *p* < 0.05 vs*.* 0.1% DDC group. Values were expressed as mean ± SD.

As shown in immunohistochemical staining (Figures 7A,B, Supplementary Figures S2 A,B), little or no α-SMA, CK19, IL-1β and IL-6 are expressed in the normal group, while a large amount of α-SMA, CK19, IL-1β and IL-6 are expressed in the model group. The positive areas of α-SMA, CK19, IL-1β and IL-6 are significantly increased in the model group (*p* < 0.01), and significantly attenuated in the astragaloside I, cycloastragenol and ASTs groups (*p* < 0.01 or *p* < 0.05, Figure 7C, Supplementary Figures S2 C,D).

qRT-PCR results show that the mRNA expressions of CK19, CK7, α-SMA and Col1A1 are significantly reduced in the astragaloside I, cycloastragenol and ASTs groups compared with the model group. And the mRNA expressions of CK7 and Col1A1 are also significantly reduced in the astragaloside II group (*p* < 0.01 or *p* < 0.05) (Figure 7D).

## Discussion

Cholestatic liver disease is a chronic liver disease that causes liver damage and fibrosis owing to bile stasis. (Karlsen et al., 2014). Ductular reaction and liver fibrosis are typical pathological manifestations of cholestatic liver disease (Fricker and Lichtenstein, 2019). Ursodeoxycholic acid (UDCA) is currently the first-line clinical drug of choice, but fails to improve disease progression at a later stage (Lindor et al., 2009). Clinical study has found that the combination of fibrates and UDCA improved serum biochemistry indices in CLD patients who responded poorly to UDCA, but had little or no effect on inhibition of disease progression (Lemoinne et al., 2018). Since no single drug or treatment has been proven to prolong transplant-free survival in CLD patients, finding a new drug has clinical values.

Recent studies have found Huangqi decoction (the main ingredient is AR) could alleviate chronic cholestatic liver injury in DDC-induced mice by improving intestinal microbiota dysbiosis and inhibiting bile acid-stimulated inflammation (Li et al., 2019; Zou et al., 2021). And Huangqi decoction also can reduce bile duct hyperplasia, and relieve liver fibrosis in bile duct ligation (BDL) rats. The mechanism may be related to regulate Numb expression and inhibit Notch signaling pathway (Zhang X. et al., 2017; Xu et al., 2020). But these studies mainly focus on the effective mechanism, without identifying the active pharmaceutical constituents of AR. In this study, ductular reaction and liver fibrosis are able to dose-dependently improved in DDC-induced CLD mice after ASTs treatment. Cholestatic liver injury is often accompanied by significant bile duct hyperplasia in the protal area (Tacke and Zimmermann, 2014). Bile duct hyperplasia is a compensatory response of the liver in response to impaired bile acid circulation. CK19 and CK7 are specific biomarkers of biliary epithelial cells (Mishra et al., 2009). And during the pathogenesis of liver fibrosis, activated hepatic stellate cells and fibroblasts express α-SMA, synthesize collagen and produce extracellular matrix (Parola and Pinzani, 2019; Zahedifard et al., 2020). The expressions of CK19 and α-SMA in liver tissues of CLD mice are significantly decreased after continuous oral administration of ASTs for 4 weeks. These results suggest that ASTs has a significant improvement effect on CLD mice.

UHPLC-Q-Orbitrap Exactive HRMS system is now widely used for the identification and quantitative analysis of components in TCM or compound prescriptions (Liu et al., 2021; Zhang C. et al., 2022). In this study, 45 kinds of astragalus saponins are identified in ASTs by UHPLC-Q-Orbitrap Exactive HRMS technology, seven kinds of astragalus saponins are identified by standards, including astragaloside I, astragaloside II, astragaloside III, astragaloside IV, isoastragaloside I, isoastragaloside II and cycloastragenol. And astragaloside I, astragaloside II, astragaloside IV and cycloastragenol are found in mice serum or liver tissues after oral administration of ASTs. Next, the transdifferentiation of WB-F344 cell model induced by SB is to evaluate the effects of ASTs and main components (Wang et al., 2010). qRT-PCR results revealed that the expression of CK19 in SB-induced WB-F344 cell is significantly decreased in these seven saponins constituents treatment groups. Astragaloside I can be sequentially converted to astragaloside II, astragaloside IV and cycloastragenol in rats after oral administration of AR (Li et al., 2017). Consistent with this, a little astragaloside I is found in the serum and liver tissues in our experiments, it has probably converted to other saponins *in vivo*. Combining the results of the absorbed chemical analysis *in vivo* and the activity screening *in vitro* experiments, astragaloside I, astragaloside II, astragaloside IV and cycloastragenol are finally selected, and the improvement effects of them are evaluated on cholestatic liver disease mouse model.

**FIGURE 8:**
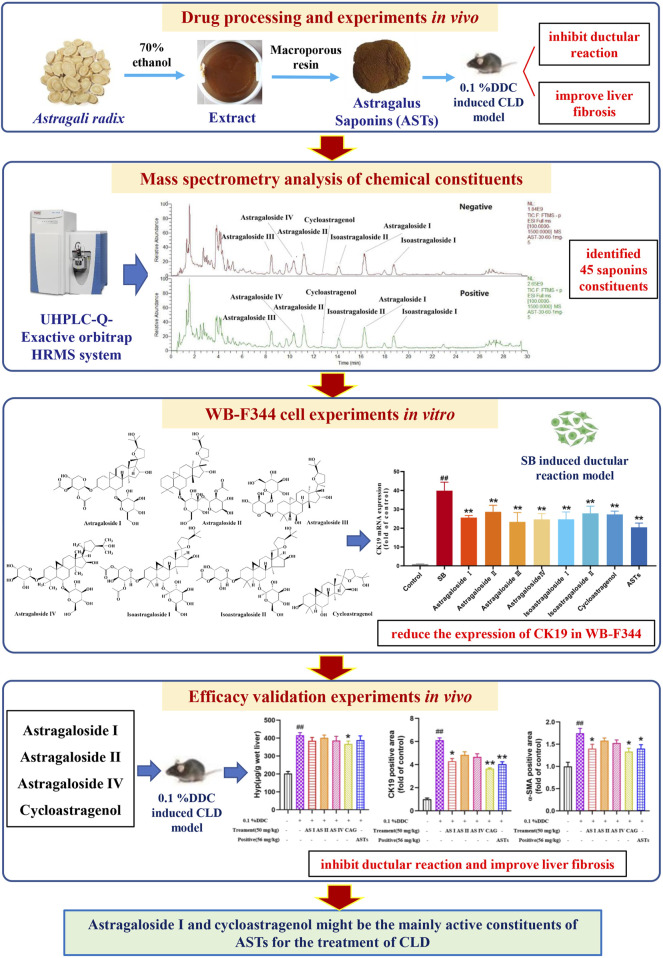
Scheme of the study. The main experiments in sequence include drug processing and *in vivo* experiments, mass spectrometry analysis of chemical constituents, *in vitro* WB-F344 cell experiments, and *in vivo* efficacy validation experiments.

It is found that astragaloside IV is able to inhibit liver inflammation *via* mitogen-activated protein kinase (MAPK)/nuclear factor kappa-B (NF-κB) signaling pathway, which in turn reduce lipid levels, hepatic steatosis in hyperlipidemic mice (Zhang Y. et al., 2022). Astragaloside IV significantly reduces body weight, white fat and liver/body weight ratio in senescent mice by targeting mitochondrial activity, and promotes fatty acid in white adipose tissue mobilization in aging mice (Luo et al., 2021). Cycloastragenol activates Farnesoid X receptor (FXR) to reduce hepatic lipid accumulation, blood glucose, serum triacylglycerol levels and bile acids in high-fat diet mice. And cycloastragenol also attenuates hepatic steatosis in mice with methionine choline-deficient diet-induced nonalcoholic steatohepatitis (Gu et al., 2017). However, there are few studies related to astragaloside I and astragaloside II in liver diseases, especially cholestatic liver disease. Our experimental results show that Hyp content, abnormalities of serum biochemistry and the hepatic expressions of CK19 and α-SMA in CLD mice are significantly alleviated in cycloastragenol and astragaloside I treatment groups. It is reported that AR could regulate arachidonic acid metabolism and ether lipid metabolism by modulating the expressions of cytochrome P450 1A2 (CYP1A2), phosphate Cytidylyltransferase 1A (PCYT1A) and cytochrome P450 Family one Subfamily B Member 1 (CYP1B1) through network pharmacology techniques, to effectively ameliorate liver fibrosis (Wang et al., 2019). And network pharmacology study also found that astragalus flavonoids exerted anti-fibrosis effects by inhibiting IκB kinase β (IKKβ)/NF-κB pathway (An et al., 2020). However, these network pharmacology studies have not explored in-depth to find out the active constituents, and further validation experiments were lacking. In this study, we innovatively use UHPLC-Q-Orbitrap Exactive HRMS technology, combined with *in vivo* and *in vitro* experiments to find out the active constituents of ASTs exerting the therapeutic effects in cholestatic liver disease (Figure 8). And this strategy is promising for further investigation of other TCM herbs and formulaes.

However, the relationship between different effective constituents of ASTs and the protective mechanism against cholestatic liver disease should be further studied. In additional, the second *in vivo* experiment is not able to set up multiple doses of drug concentrations, which is also a shortcoming of the present study.

## Conclusion

In this study, ASTs are found to have significant therapeutic effects on cholestatic liver disease, which are the main active site of AR against CLD, astragaloside I and cycloastragenol significantly inhibit the progression of cholestatic liver disease, which may the most important active constituents of ASTs for CLD. These findings not only provide a scientific basis for clarifying the active constituents of ASTs in the treatment of CLD, but also suggest novel promising therapeutic drugs for CLD.

## Data Availability

The original contributions presented in the study are included in the article/Supplementary Materials, further inquiries can be directed to the corresponding authors.
