# Supplementary material for: Astragalus saponins and its main constituents ameliorate ductular reaction and liver fibrosis in a mouse model of DDC-induced cholestatic liver disease
Source: Front Pharmacol. 2022 Oct 20;13:965914. doi: 10.3389/fphar.2022.965914 (PMC9632275; doi:10.3389/fphar.2022.965914)
Supplement: Supplementary file 1 [file DataSheet1.docx]

**Supplementary materials**

***for***

**Astragalus saponins and its main constituents ameliorate ductular reaction and liver fibrosis in a mouse model of DDC-induced primary sclerosing cholangitis**

Linzhang Zhang ^a, b, 1^, Yonghong Hu ^a,1^**,** Shenglan Qi ^b^, Congcong Zhang ^a^, Qun Zhou ^a, b^, Dingqi Zhang ^a^, [Yongping Mu](https://pubmed.ncbi.nlm.nih.gov/?size=50&term=Mu+Y&cauthor_id=34630075) ^a^, [Hua Zhang](https://pubmed.ncbi.nlm.nih.gov/?size=50&term=Zhang+H&cauthor_id=34630075) ^a^, [Gaofeng Chen](https://pubmed.ncbi.nlm.nih.gov/?size=50&term=Chen+G&cauthor_id=33332219) ^a^, [Ping Liu](https://pubmed.ncbi.nlm.nih.gov/?size=50&term=Liu+P&cauthor_id=34630075) ^a, b^ *, [Jiamei Chen](https://pubmed.ncbi.nlm.nih.gov/?size=50&term=Chen+J&cauthor_id=34630075) ^a,^*, Wei Liu ^a,^*

*^a^Institute of Liver Diseases, Key Laboratory of Liver and Kidney Diseases (Ministry of Education), Shanghai Key Laboratory of Traditional Chinese Clinical Medicine, Shuguang Hospital Affiliated to Shanghai University of Traditional Chinese Medicine, 528 Zhangheng Road, Shanghai 201203, China*

*^b^Shanghai Frontiers Science Center of TCM Chemical Biology; Institute of Interdisciplinary Integrative Medicine Research, Shanghai University of Traditional Chinese Medicine, Shanghai, 201203, China*

^1^Linzhang Zhang and Yonghong Hu contributed equally to this work.

*Correspondence to: Associate Professor Wei Liu, [Jiamei Chen](https://pubmed.ncbi.nlm.nih.gov/?size=50&term=Chen+J&cauthor_id=34630075), and Professor Ping Liu, Institute of Liver Diseases, Key Laboratory of Liver and Kidney Diseases (Ministry of Education), Shanghai Key Laboratory of Traditional Chinese Clinical Medicine, Shuguang Hospital Affiliated to Shanghai University of Traditional Chinese Medicine, Shanghai 201203, China. lwhzayl@163.com (W. Liu), [cjm0102@126.com](mailto:cjm0102@126.com) (J M. Chen) and liuliver@vip.sina.com (P. Liu)

***1. Effects of different astragalus saponins on the expressions of biliary senescence***

***in 0.1 % DDC-induced CLD mice***


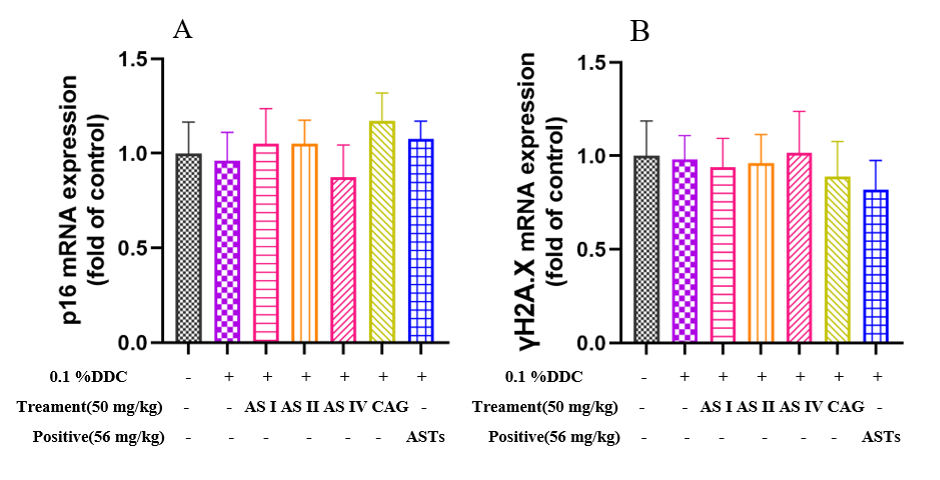


**FIGURE S1**  **Effects of different astragalus saponins on the expressions of biliary senescence in 0.1 % DDC-induced CLD mice.** (A, B) Effects of different astragalus saponins on the gene expressions of p16 and γH2A.x are evaluated by qRT-PCR, and normalized to GAPDH gene. ##*p* < 0.01 vs the normal group. ∗∗*p* < 0.01 and ∗*p* < 0.05 vs 0.1 % DDC group. Values were expressed as mean ± SD.

***2. Astragaloside I and Cycloastragenol Supppressed the Expressions of IL-1β and IL-6 in DDC-Induced PSC Mice***

***
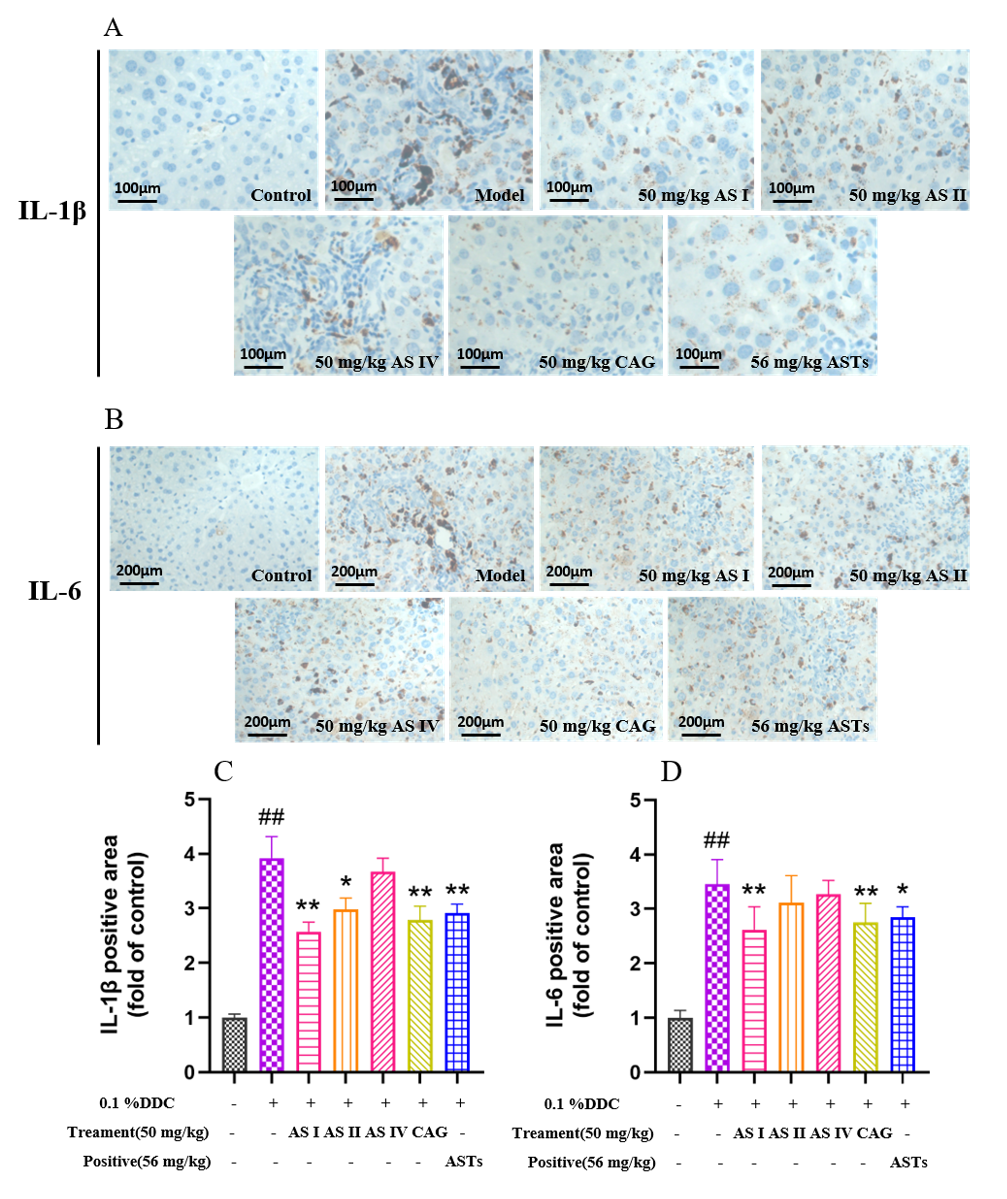
***

**FIGURE S2 Effects of astragaloside I, astragaloside II, astragaloside IV and cyclogalactol on the expressions of pro-inflammatory cytokines in 0.1 % DDC-induced CLD mice.** (A, B) Immunohistochemical method is used to detect the effects of different astragalus saponins on hepatic IL-1β and IL-6 protein expressions (×20x, ×10x) (scale bar 100 μm, 200 μm). (C, D) Morphometric quantification of the IL-1β and IL-6 positive area (%). ##*p* < 0.01 vs the normal group. ∗∗*p* < 0.01 and ∗*p* < 0.05 vs 0.1 % DDC group. Values were expressed as mean ± SD.

***3. Chromatography conditions for UHPLC-Q-Exactive Orbitrap HRMS***

Chromatographic separation is performed on an UHPLC-Q-Exactive Orbitrap HRMS system (Thermo Fisher Scientific Inc., Grand Island, NY, USA). The UHPLC system consisted of a Thermo Scientific Dionex Ultimate 3000 Series RS pump coupled with a Thermo Scientific Dionex Ultimate 3000 Series TCC-3000RS column compartments and WPS-3000 autosampler controlled by Chromeleon 7.2 Software. The cooling autosampler is set at 4 °C and protected from light, and the column heater is set at 40 °C. A Waters ACQUITY UPLC BEH C_18_ column (2.1 mm × 100 mm, 1.7 μm) is employed with the temperature set at 40 °C. The mobile phase consists of A (methanol) and B (0.1% formic acid) at a flow rate of 0.3 mL·min-1 and eluts with gradient elution: 0-1 min (40% A), 1-2 min (60%-65% A), 2-10 min (65% A), 10-24 min (65%-75% A), 24-25 min (75%-95% A), 25-28 min (95% A), 28-30 min (60% A). The injection volume is 2 μL.

The mass spectrometer Q-Exactive Orbitrap system is connected to the UHPLC system *via* heated electrospray ionization and controlled by Xcalibur 4.1 software that is used for data collection and analysis. The electrospray ionization source is operated and optimized in negative and positive ionization mode. The optimized parameters of mass spectrometry are: capillary temperature: 325 °C; sheath gas (N_2_) flow rate: 45 arbitrary units; auxiliary gas (N_2_) flow rate: 8 arbitrary units; sweep gas flow rate: 0 arbitrary units; spray voltage: 2.5 kV (negative), 3.5 kV (positive); S-lens RF level: 50V; auxilliary gas heater temperature, 300 °C; scan mode: Full MS/SIM and Full MS/dd-MS^2^ mode, which includes 1 first-level full scan (resolution 70000 FWHM) and 1 data-dependent secondary scan (resolution 17500 FWHM) 2 events, the scanning range is 80-1200 *m*/*z*, and the collision energy gradient is 20, 50, 100V.

**Table S1 Ingredient identification of AST from Astragali radix by UHPLC-Q-Exactive Orbitrap HRMS**

| No. | RT /min | Ion mode | Measured mass /Da | Calculated mass /Da | Error /ppm | Molecular formula | Fragment ions | Identification | Parent nucleus | Peak Area (*10^6^)  AST Sreum Liver | | |
| --- | --- | --- | --- | --- | --- | --- | --- | --- | --- | --- | --- | --- |
| 1 | 5.64 | [M+HCOO]^-^ | 781.4370 | 781.4369 | 0.067 | C_40_H_64_O_12_ | 783.4522; 596.6781; 489.3582; 433.6416 | Huangqiyenins F | Type V | 2.30 | - | - |
| 2 | 6.13 | [M+HCOO]^-^ | 699.4248 | 699.4314 | -6.604 | C_36_H_62_O_10_ | 463.4926; 340.9177; 91.9471 | Alexandroside Ⅰ | Type III | 5.54 | - | - |
| 3 | 6.53 | [M+HCOO]^-^ | 697.4075 | 697.4158 | -0.399 | C_36_H_60_O_10_ | 697.4187; 508.4275 | AstraverrucinⅠ | Type I | 5.92 | - | - |
| 4 | 6.58 | [M-H]^-^ | 669.4235 | 669.4208 | 2.681 | C_36_H_62_O_11_ | 654.3877;491.3735 | Mongholicoside A | Type II | 51.36 | - | - |
| 5 | 6.71 | [M+HCOO]^-^ | 697.4074 | 697.4158 | -0.519 | C_36_H_60_O_10_ | 697.4181; 634.4705; 455.6770; 218.4356 | Huangqiyenins B | Type II | 16.97 | - | - |
| 6 | 6.92 | [M-H]^-^ | 953.4772 | 953.4741 | 3.114 | C_48_H_74_O_19_ | 953.4758; 732.6904; 627.3587;469.3320 | Malonylastragaloside I | Type I | 21.87 | - | - |
| 7 | 7.01 | [M-H]^-^ | 669.4238 | 669.4208 | 2.931 | C_36_H_62_O_11_ | 669.4238; 491.3744 | Mongholicoside B | Type II | 18.52 | - | - |
| 8 | 7.20 | [M+HCOO]^-^ | 991.5136 | 991.5108 | 2.774 | C_47_H_78_O_19_ | 945.5081; 783.4516; 621.3995; 453.5482 | Astragaloside VII | Type I | 140.51 | - | - |
| 9 | 7.46 | [M+H]^+^ | 459.3829 | 459.3833 | -0.402 | C_30_H_50_O_3_ | 459.3816; 441.3725; 203.1795; 189.1636; 151.1118; 109.1016 | Soyasaponin B | Type IV | 10.80 | - | - |
| 10 | 8.14 | [M+HCOO]^-^ | 805.4342 | 805.4518 | 2.402 | C_40_H_62_O_15_ | 805.4542; 727.2454; 403.5289 | Huangqiyenins I | Type VI | 3.37 | - | - |
| 11 | 8.23 | [M+HCOO]^-^ | 803.4031 | 803.4005 | 2.583 | C_40_H_60_O_13_ | 661.4630; 530.0955; 303.5872 | Huangqiyenins H | Type V | 4.39 | - | - |
| 12 | 8.29 | [M+HCOO]^-^ | 829.4612 | 829.4580 | 3.228 | C_41_H_68_O_14_ | 783.4550; 621.4053; 489.3593 | Isoastragaloside IV | Type I | 102.37 | - | - |
| 13 | 8.39 | [M+HCOO]^-^ | 1075.5346 | 1075.5320 | 2.585 | C_51_H_82_O_21_ | 945.5104;927.4965;909.4866;765.4445 | Agroastragaloside Ⅲ | Type I | 55.70 | - | - |
| 14 | 9.10 | [M+HCOO]^-^ | 991.5137 | 991.5108 | 2.894 | C_47_H_78_O_19_ | 945.5081; 783.4555; 651.4123; 489.3586 | Astragaloside V | Type I | 579.91 | - | - |
| 15 | 9.71 | [M+HCOO]^-^ | 829.4610 | 829.4580 | 2.988 | C_41_H_68_O_14_ | 783.4550;621.4053; 489.3593 | Astragaloside III | Type I | 1624.72 | - | - |
| 16 | 10.14 | [M+HCOO]^-^ | 943.5192 | 943.5261 | -6.922 | C_47_H_78_O_16_ | 943.4030; 717.4015 | Isoastragaloside VI | Type I | 64.70 | - | - |
| 17 | 10.14 | [M-H]^-^ | 941.5129 | 941.5104 | 2.498 | C_48_H_78_O_18_ | 941.5132; 615.3905; 457.3685; | Soyasaponin Bb | Type IV | 1017.52 | 89.57 | 97.54 |
| 18 | 10.20 | [M-H]^-^ | 987.5184 | 987.5159 | 2.449 | C_49_H_80_O_20_ | 941.5132;924.5035;795.4599; 733.4520; 615.3917 | Agroastragaloside Ⅳ | Type I | 244.53 | - | - |
| 19 | 10.28 | [M+HCOO]^-^ | 695.4033 | 695.4001 | 3.216 | C_36_H_58_O_10_ | 666.4238; 541.4501; 295.2294 | Huangqiyenins A | Type I | 6.42 | - | - |
| 20 | 10.32 | [M+HCOO]^-^ | 829.4607 | 829.4580 | 2.678 | C_41_H_68_O_14_ | 783.4572; 757.4901; 657.3870; 489.3607; 448.2976 | Astragaloside IV | Type I | 2055.83 | 8060.30 | 9604.17 |
| 21 | 10.37 | [M+HCOO]^-^ | 795.4168 | 795.4162 | 0.782 | C_40_H_62_O_13_ | 795.4555; 615.3816 | Huangqiyenins G | Type V | 1.93 | - | - |
| 22 | 10.49 | [M-H]^-^ | 911.5025 | 911.4999 | 2.263 | C_47_H_76_O_17_ | 911.5020; 893.4878; 615.3895; 571.3978; 457.3688 | Astragaloside VIII | Type IV | 201.20 | - | - |
| 23 | 10.69 | [M+Na]^+^ | 791.4157 | 791.4134 | 2.362 | C_40_H_60_O_14_ | 791.4544; 719.3335; 516.6068;306.3629; 175.0600; 157.0494 | Huangqiyenins J | Type V | 8.42 | - | - |
| 24 | 11.20 | [M+Na]^+^ | 809.4670 | 809.4658 | 1.202 | C_41_H_70_O_14_ | 437.3403;341.2481 | Astramembranosides B | Type II | 205.63 | - | - |
| 25 | 11.23 | [M+HCOO]^-^ | 871.4711 | 871.4686 | 2.493 | C_43_H_70_O_15_ | 871.4713; 413.3819; 250.3977; 113.02338; 101.0228 | Astragaloside II | Type I | 4176.41 | 7437.46 | 8941.81 |
| 26 | 11.23 | [M+HCOO]^-^ | 873.4772 | 873.4842 | -6.997 | C_43_H_72_O_15_ | 705.4680; 405.3112 | Agroastragaloside ⅠI | Type II | 637.13 | - | - |
| 27 | 11.28 | [M+HCOO]^-^ | 697.4069 | 697.4158 | -1.009 | C_36_H_60_O_10_ | 697.4184; 337.4438 | Brachyosides B | Type I | 146.43 | - | - |
| 28 | 11.43 | [M+Na]^+^ | 645.3967 | 645.3973 | -0.634 | C_35_H_58_O_9_ | 645.3968; 304.5002 | Atramembrannin | Type I | 197.44 | - | - |
| 29 | 11.64 | [M+Na]^+^ | 513.3546 | 513.3551 | -0.496 | C_30_H_50_O_5_ | 513.3546; 359.4659; 195.2986 | Isoastragaloside VII | Type I | 2.20 | - | - |
| 30 | 12.95 | [M+Na]^+^ | 513.3549 | 513.3551 | -0.126 | C_30_H_50_O_5_ | 513.3549; 466.2076 | Cycloastragenol | Type I | 60.63 | 2387.77 | 3410.48 |
| 31 | 13.21 | [M+Na]^+^ | 513.3516 | 513.3551 | -0.328 | C_30_H_50_O_5_ | 513.3546;306.5692;164.5694 | Huangqiyenins Ⅱ | Type II | 3.17 | - | - |
| 32 | 13.72 | [M+H]^+^ | 489.3571 | 489.3575 | -0.196 | C_30_H_48_O_5_ | 489.3558;319.4040;250.8706 | Huangqiyenins Ⅰ | Type I | 8.63 | - | - |
| 33 | 14.12 | [M+HCOO]^-^ | 943.5194 | 943.5261 | -6.742 | C_47_H_78_O_16_ | 943.4017; 608.3420; 265.6801; 116.9272 | Astragaloside VI | Type I | 176.90 | - | - |
| 34 | 14.14 | [M+HCOO]^-^ | 871.4714 | 871.4686 | 2.793 | C_43_H_70_O_15_ | 871.4711; 808.4833; 709.5390; 421.3489 | Isoastragaloside II | Type I | 1752.16 | - | - |
| 35 | 14.15 | [M+Na]^+^ | 809.4674 | 809.4658 | 1.572 | C_41_H_70_O_14_ | 455.3528; 437.3413; 401.3189; 297.2213 | Cyclocanthosides E | Type II | 90.32 | - | - |
| 36 | 16.20 | [M+Na]^+^ | 817.4335 | 817.4345 | -0.948 | C_42_H_66_O_14_ | 817.4350;760.4130; 381.5313 | Huangqiyenins E | Type V | 11.50 | - | - |
| 37 | 16.26 | [M+HCOO]^-^ | 913.4820 | 913.4791 | 2.848 | C_45_H_72_O_16_ | 913.4818; 510.3442; 448.2912; 243.4432; 113.02318; 101.0230 | Astragaloside I | Type I | 4287.52 | 507.46 | 634.51 |
| 38 | 16.31 | [M+Na]^+^ | 835.4747 | 835.4814 | -6.758 | C_43_H_72_O_14_ | 833.4672; 707.3795; 419.3314 | AgroastragalosideⅤ | Type II | 33.77 | - | - |
| 39 | 17.26 | [M+HCOO]^-^ | 915.4881 | 915.4948 | -6.692 | C_45_H_74_O_16_ | 915.4979;747.7819;410.2894;212.4037 | Agroastragaloside Ⅰ | Type II | 690.03 | - | - |
| 40 | 17.37 | [M-H]^-^ | 813.4661 | 813.4631 | 2.962 | C_42_H_70_O_15_ | 767.4609; 586.4556; 473.3627 | Astramembranosides A | Type I | 57.38 | - | - |
| 41 | 17.96 | [M+Na]^+^ | 809.4672 | 809.4658 | 1.382 | C_41_H_70_O_14_ | 730.4824; 456.3508; 437.3416; 364.6031; 297.2216 | Isocyclocanthosides E | Type III | 57.38 | - | - |
| 42 | 18.71 | [M+Na]^+^ | 835.4735 | 835.4814 | -7.918 | C_43_H_72_O_14_ | 817.8998;419.3310 | Isoastragaloside V | Type I | 69.95 | - | - |
| 43 | 18.74 | [M+HCOO]^-^ | 913.4816 | 913.4791 | 2.608 | C_45_H_72_O_16_ | 913.4831; 729.3085; 338.3886;187.3476 | Isoastragaloside I | Type I | 1955.75 | - | - |
| 44 | 22.93 | [M+HCOO]^-^ | 9134822 | 913.4791 | 3.098 | C_45_H_72_O_16_ | 913.4831; 411.45285; 352.4134; 113.0228 | Neoastragaloside I | Type I | 422.42 | - | - |
| 45 | 24.77 | [M+Na]^+^ | 933.4809 | 933.4818 | -0.922 | C_47_H_74_O_17_ | 933.4812;753.4117 | Acetytastragaloside | Type I | 17.44 | - | - |

RT-retention time. “-“ was not detect.
